# A noninvasive method for predicting clinically significant prostate cancer using magnetic resonance imaging combined with PRKY promoter methylation level: a machine learning study

**DOI:** 10.1186/s12880-024-01236-1

**Published:** 2024-03-11

**Authors:** Yufei Wang, Weifeng Liu, Zeyu Chen, Yachen Zang, Lijun Xu, Zheng Dai, Yibin Zhou, Jin Zhu

**Affiliations:** 1 Department of Urology, The Second Affiliated Hospital of Soochow University, Suzhou, Jiangsu Province 215000 China; 2 Department of Urology, Hefei First People’s Hopital, Hefei, Anhui Province 230000 China

**Keywords:** Radiomics, Clinically significant prostate cancer, PRKY promoter methylation, Machine learning

## Abstract

**Background:**

Traditional process for clinically significant prostate cancer (csPCA) diagnosis relies on invasive biopsy and may bring pain and complications. Radiomic features of magnetic resonance imaging MRI and methylation of the PRKY promoter were found to be associated with prostate cancer.

**Methods:**

Fifty-four Patients who underwent prostate biopsy or photoselective vaporization of the prostate (PVP) from 2022 to 2023 were selected for this study, and their clinical data, blood samples and MRI images were obtained before the operation. Methylation level of two PRKY promoter sites, cg05618150 and cg05163709, were tested through bisulfite sequencing PCR (BSP). The PI-RADS score of each patient was estimated and the region of interest (ROI) was delineated by 2 experienced radiologists. After being extracted by a plug-in of 3D-slicer, radiomic features were selected through LASSCO regression and t-test. Selected radiomic features, methylation levels and clinical data were used for model construction through the random forest (RF) algorithm, and the predictive efficiency was analyzed by the area under the receiver operation characteristic (ROC) curve (AUC).

**Results:**

Methylation level of the site, cg05618150, was observed to be associated with prostate cancer, for which the AUC was 0.74. The AUC of T2WI in csPCA prediction was 0.84, which was higher than that of the apparent diffusion coefficient ADC (AUC = 0.81). The model combined with T2WI and clinical data reached an AUC of 0.94. The AUC of the T2WI-clinic-methylation-combined model was 0.97, which was greater than that of the model combined with the PI-RADS score, clinical data and PRKY promoter methylation levels (AUC = 0.86).

**Conclusions:**

The model combining with radiomic features, clinical data and PRKY promoter methylation levels based on machine learning had high predictive efficiency in csPCA diagnosis.

## Background

Prostate cancer (PCa) is the second most common cancer among male cancers worldwide and the leading cause of death owing to cancer of men in 46 countries [1]. The International Society of Urological Pathology (ISUP) Grading Group system is significant for deciding on the treatment for PCa. Recently, some studies have supported reconsidering the classification of ISUP Grade 1 PCa (so called non-clinically significant PCa, ncsPCA) because its risk is closer to that of benign prostatic hyperplasia (BPH) to avoid overtreatment [2, 3]. For ISUP Grade ≥ 2 PCa (so called clinically significant PCa, csPCA) patients, a timely treatment is necessary, while current guidelines mainly recommend active surveillance rather than radical prostatectomy for ncsPCA patients [4]. To separate csPCA patients from ncsPCA and BPH patients for prompt intervention, prostate biopsy is necessary, which brings pain, complications and more medical costs to patients [5–7]. Some early studies showed that about 30% of patients reported significant pain, and 16% patients reached a score over or equal to five on the pain visual analog scale of one to ten [8, 9]. As common complications after biopsy, bleeding and infection may also be fetal to patients especially when massive rectal bleeding or septicemia happens [10]. A simplified procedure for csPCA diagnosis is needed to address the issue.

PRKY is a pseudogene as the homolog gene of PRKX on human Y chromosome, which is related to the Xp;Yp translocation and is reportedly associated with testicular disorder of sex development and infertility [11–14]. Previous studies have proposed a correlation between the methylation of a site, cg05618150, on the PRKY promoter and PCa, suggesting the potential of PRKY promoter methylation as a new biomarker participating in the prediction of csPCA [15]. Our previous research has found another methylation site, cg05618150, is also associated with PCa.

Magnetic resonance imaging (MRI) has been recommended in recent guideline for its high value for PCa diagnosis [4]. Traditional imaging interpretation based on the prostate imaging reporting and data system (PI-RADS) score system highly relies on the experience and capacity of radiologists [16, 17]. The Radiomics refers to a subject that use image features to predict the diagnosis or even the prognosis of diseases [18]. Radiomic models have been proved to be effective in diagnosing urinary tumors. Durgesh et al. has made a model combined with MRI features with high predictive value for high-grade histology in clear cell renal cell carcinoma [19]. Another study also extracted MRI features to predict the diagnosis of muscle invasion of bladder cancer [20]. By using radiomic methods and machine learning technologies, imaging features can be extracted and their associations with tumor attributes can be analyzed by computer programs, hence, the variation between imaging readers can be partly controlled, and the ISUP stage of PCa can also be predicted precisely [21–23]. Therefore, it is possible to use MRI features to predict the diagnosis of csPCA.

To find a more efficient way to diagnose csPCA and avoid unnecessary biopsy, in our study, we collected the methylation data for two sites on the PRKY promoter (cg05618150 and cg05163709) in blood samples and MRI data from csPCA and ncsPCA or benign prostatic hyperplasia patients to determine the combined relevance of these data with PCa of different ISUP grades. A prediction model was constructed based on machine learning to optimize the procedure for diagnosing csPCA.

## Methods

### Patient population

**Fig. 1:**
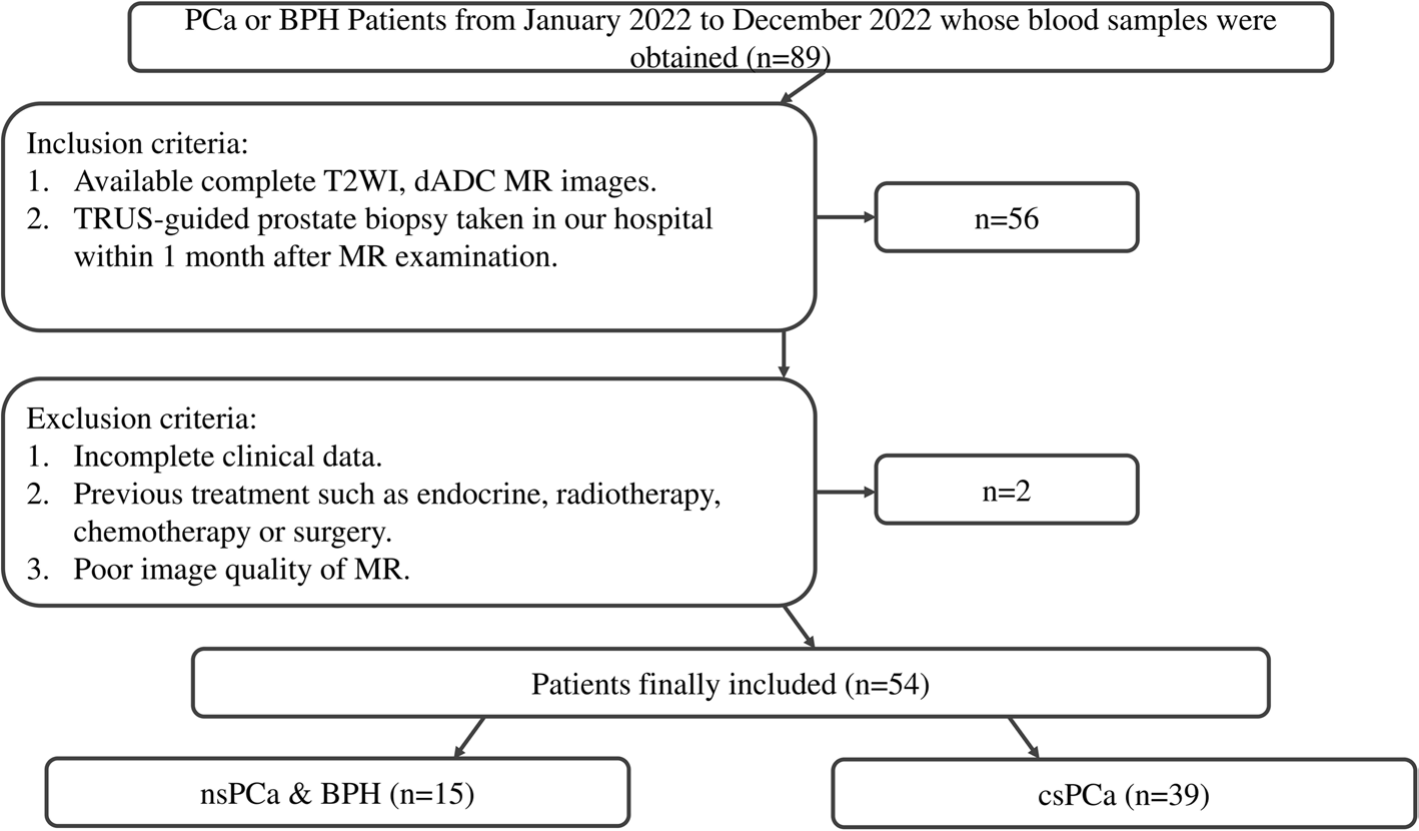
The inclusion and exclusion flow. Legend: This picture showed the inclusion and exclusion criteria of our study

This study was approved by the institutional review board of our hospital. All the patients were informed the research and signed informed consents. Eighty-nine Patients who underwent prostate biopsy or photoselective vaporization of the prostate (PVP) from 2022 to 2023 were included, and their blood samples were obtained for testing PRKY methylation levels. The detailed inclusion and exclusion criteria are exhibited in Fig. 1. According to the criteria, 54 patients were eligible for this study; 2 were excluded and 54 patients were ultimately included.

### Sample collection and DNA methylation detection

Blood samples were obtained from patients before prostate biopsy or PVP and centrifuged within 4 h after collection (centrifugal radius, 9 cm; centrifugal speed, 63.5 r/minutes; centrifugal force, 4000 g; centrifugal time, 10 min; the centrifuge was from Eppendor, Germany). Bisulfite sequencing polymerase chain reaction (BSP) was used for methylation sequencing. Two sites of PRKY promoter, cg05618150 and cg05163709, were detected by designed DNA probes (Zhengze, China) after bisulfite conversion. The β-actin sequence was selected as the control. The premixed solution was made according to Table 1. Briefly, 15 µl of solution and 10 µl of template were mixed in each well of 96-well plates and prepared for quantitative real-time polymerase chain reaction (qPCR). The qPCR instrument (Thermo Fisher, America) was set as follows: 95 °C for 5 s for initial denaturation; 95 °C for 15 s for denaturation; and 56 °C for 1 min for annealing, extension and fluorescent detection. The cycle threshold (Ct) was recorded for analysis.

**Table 1:** Elements of the premixed solution in qPCR

Name	Volume (uL)	Name	Volume (Ul)
qPCR mix	12.5	qPCR mix	12.5
Ppmix	2.5	F-cg05163709	0.3
		R-cg05163709	0.3
		P-cg05163709	0.2
		F-cg05618150	0.3
		R-cg05618150	0.3
		P-cg05618150	0.2
		F-ACTB	0.25
		R-ACTB	0.25
		P-ACTB	0.15
		ddH2O	0.25
total	15	total	15

### MR imaging

A 3.0-T MR scanner (Ingenia; Philips Healthcare) was used for image acquisition. The parameters of T2-weighted imaging (T2WI) sequences were as follows: flip angle (FA): 90°; repetition time (TR): 3000 ms; echo time (TE): 100 ms; number of excitations (NEX): 2; slice thickness: 3 mm; field of view (FOV): 260 mm; and acquisition matrix: 560 × 560. Diffusion-weighted imaging (DWI) images were obtained by using echoplanar imaging sequences, the parameters were shown as follows: FA: 90°; TR: 6500 ms; TE: 65 ms; NEX: 2; FOV: 260 mm; and acquisition matrix: 224 × 224. Apparent diffusion coefficient (ADC) maps were got from a designed workstation in our hospital. All the images were obtained in the DICOM format.

### Pathology status

For all patients, transrectal ultrasonography guided prostate biopsy or greenlight PVP were performed within 1 month after MRI examination. After that, all tissue specimens were labeled according to patient ID and analyzed by an experienced pathologist. (The Gleason score of each specimen was checked by another experienced pathologist. For patients with various Gleason scores, the highest score was recorded for grouping).

### ROI segmentation

MRI images were reviewed by two radiologists specializing in abdominal MRI for more than 10 years. An open-source software (3D-Slicer, version 5.2.2) was used for region of interest (ROI) segmentation. The regions with low signal intensity on T2WI and ADC as well as high signal intensity on DWI were regarded as the tumor areas, and the PI-RADS score of each tumor area was estimated. For tumors with various foci, the regions with the highest PI-RADS score were selected for analysis. After one radiologist had completed the evaluation and 3D segmentation, the other radiologist checked the results. All the differences were resolved through discussion and reexamination.

### Feature extraction and selection

The intensity features were extracted through a plug-in for 3D-Slicer (PyRadiomics, version 3.0.1). A total of 122 features were extracted from each patient in a single sequence, of which 18 were first-order statistics, 14 were shape features, 15 belonged to original features and 75 belonged to texture features. The texture features included 24 Gy-level cooccurrence matrix (GLCM) features, 14 Gy-level dependence matrix features, 16 Gy-level run length matrix (GLRLM) features, 16 Gy-level size zone matrix (GLSZM) features and 5 neighboring gray tone difference matrix (NGTDM) features. Thus, a total of 366 features were extracted from T2WI sequence, ADC (b = 100–1000) and ADC (b = 100–2000) of each patient. Feature selection and dimensionality reduction were completed by t test and LASSCO regression on the Python platform.

### Statistical analysis

**Fig. 2:**
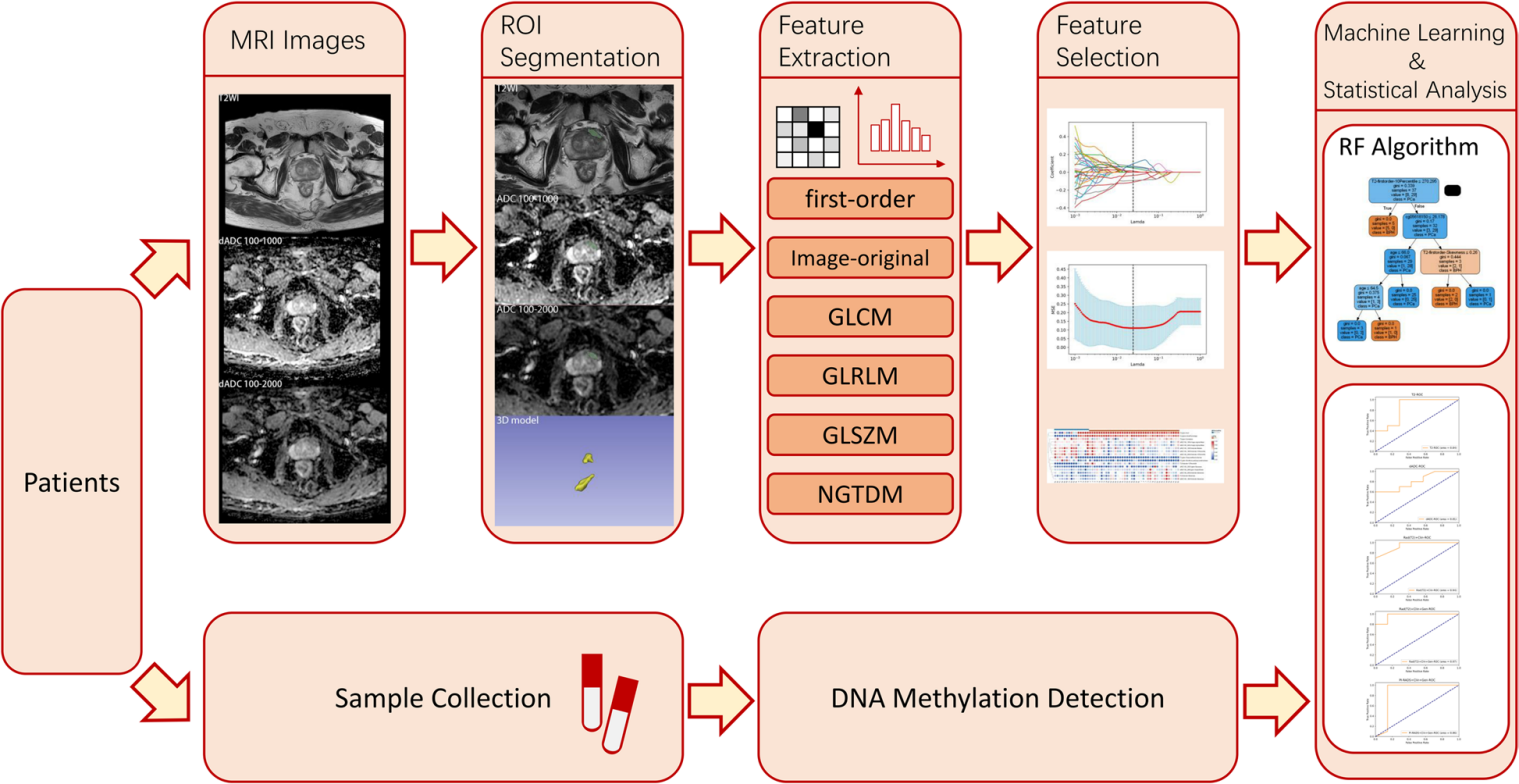
The workflow of our study. Legend: This picture exhibited the work flow of our study, including sample collection, DNA methylation detection, imaging obtainment, feature extraction and statistical analysis

R-4.2.3 (https://www.r-project.org/) was used for statistical analysis of clinical and methylation data, and *p*-value < 0.05 was regarded as statistically significant. The normality test was performed through the Shapiro–Wilk test. The t-test was used for homogeneity comparisons of normally distributed variables, and the Mann–Whitney test was used for non-normally distributed variables. We used the random forest (RF) algorithm to construct predictive models on the Python platform. All the models went through cross-validation. The receiver operation characteristics (ROC) curve and the area under the ROC curves (AUC) were used for predictive efficiency assessment. The entire workflow of this study is shown in Fig. 2.

## Results

### Patient characteristics

Thirty-nine patients (median age, 74; range, 58–86) with csPCA and 15 patients (median age, 69; range, 58–87) with BPH or ncsPCA were included. A summary of the prostate specific antigen (PSA) levels, prostrate volumes and PI-RADS scores of the csPCA group as well as the BPH and ncsPCA group were exhibited in Table 1. No significant differences were observed in age (*p* = 0.13), PSA level (*p* = 0.15), or prostrate volume (*p* = 0.09) between these two groups. The PI-RADS score (*p* < 0.0001) differed between the two groups (Table 2).

**Table 2:** Clinical data and PI-RADS score of csPCA and nsPCA or BPH patients

		nsPCA&BPH (*n* = 15)	csPCA (*n* = 39)	*P* value
Age(mean/range)		70.20(58/87)	73.51(58/86)	0.1252
PSA(ng/mL)(median/range)		9.35(3.75/90.5)	14.3(0.014/2505)	0.1489
Prostate volume(mL)(median/range)		64.4(33.4/135.9)	52.2(23/124.7)	0.0924
PI-RADS socre (num/percentage)				< 0.0001
	< 3	8(53.33)	0(0.00)	
	3	3(20.00)	7(17.95)	
	≥ 4	4(26.67)	32(82.05)	

### PRKY promoter methylation

**Fig. 3:**
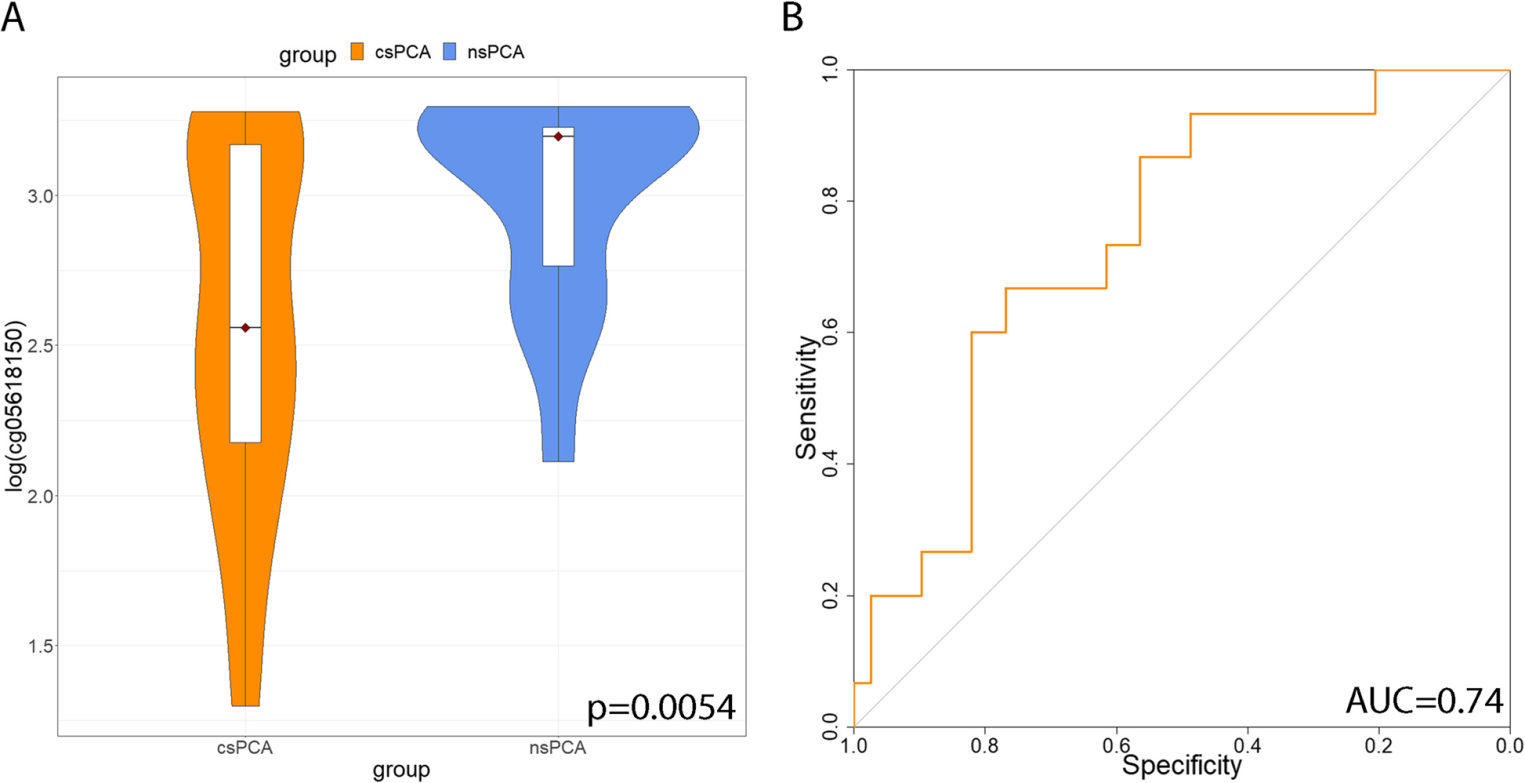
The data distribution and ROC curve of the cg05618150 methylation level. Legend: **A** Data distribution of the cg05618150 methylation level in the csPCA and ncsPCA groups. **B** ROC curve of cg05618150 methylation level to predict csPCA

After PCR, the difference in Ct between the sample and control (ΔCt) was calculated. There was no difference in the methylation level of cg05163709 between the csPCA and ncsPCa groups (*p* = 0.46). With regard to cg05618150, a lower ΔCt could be observed in the csPCa group than in the ncsPCa group (Fig. 3), which suggested a higher methylation level. The difference between the two groups was statistically significant (*p* = 0.0052). The ROC curve in Fig. 3 shows the predictive value of PRKY promoter methylation for csPCa (AUC = 0.74, *p* = 0.0059).

### MR feature selection

**Fig. 4:**
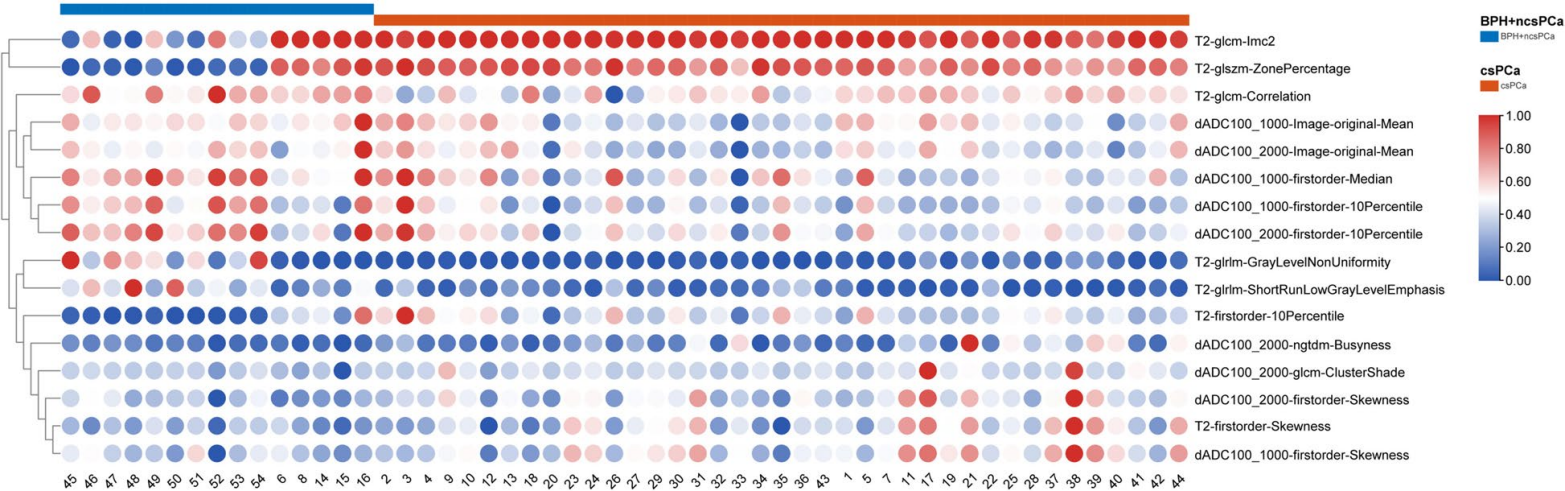
Radiomic features selected by LASSCO regression and t-test. Legend: The red dots refer to positive correlation and the blue dots refers to negative correlation

Features were extracted from T2WI and ADC images through the method described above. In total, 7 features from T2WI sequences and 9 from ADC sequences were finally selected, including T2-glcm-lmc2, T2-glszm-ZonePercentage, T2-glcm-correlation, dADC100_1000-Image-original-Mean, dADC100_2000-Image-original-Mean, dADC100_1000-firstorder-Median, dADC100_1000-firstorder-10Percentile, dADC100_2000-firstorder-10Percentile, T2-glrlm-GrayLevelNonUniformity, T2-glrlm-ShortRunLowGrayLevelEmphasis, T2-firstorder-10Percentile, dADC100_2000-ngtdm-Busyness, dADC100_2000-glcm-ClusterShade, dADC100_2000-firstorder-Skewness, T2-firstorder-Skewness and dADC100_1000-firstorder-Skewness, 7 of which are first-order statistics and 9 are texture features (3 GLCM, 2GLRLM, 1 GLSZM, 1 NGTDM and 2 image-original features). Figure 4 exhibits the matrix for all the selected features.

### Machine learning

**Fig. 5:**
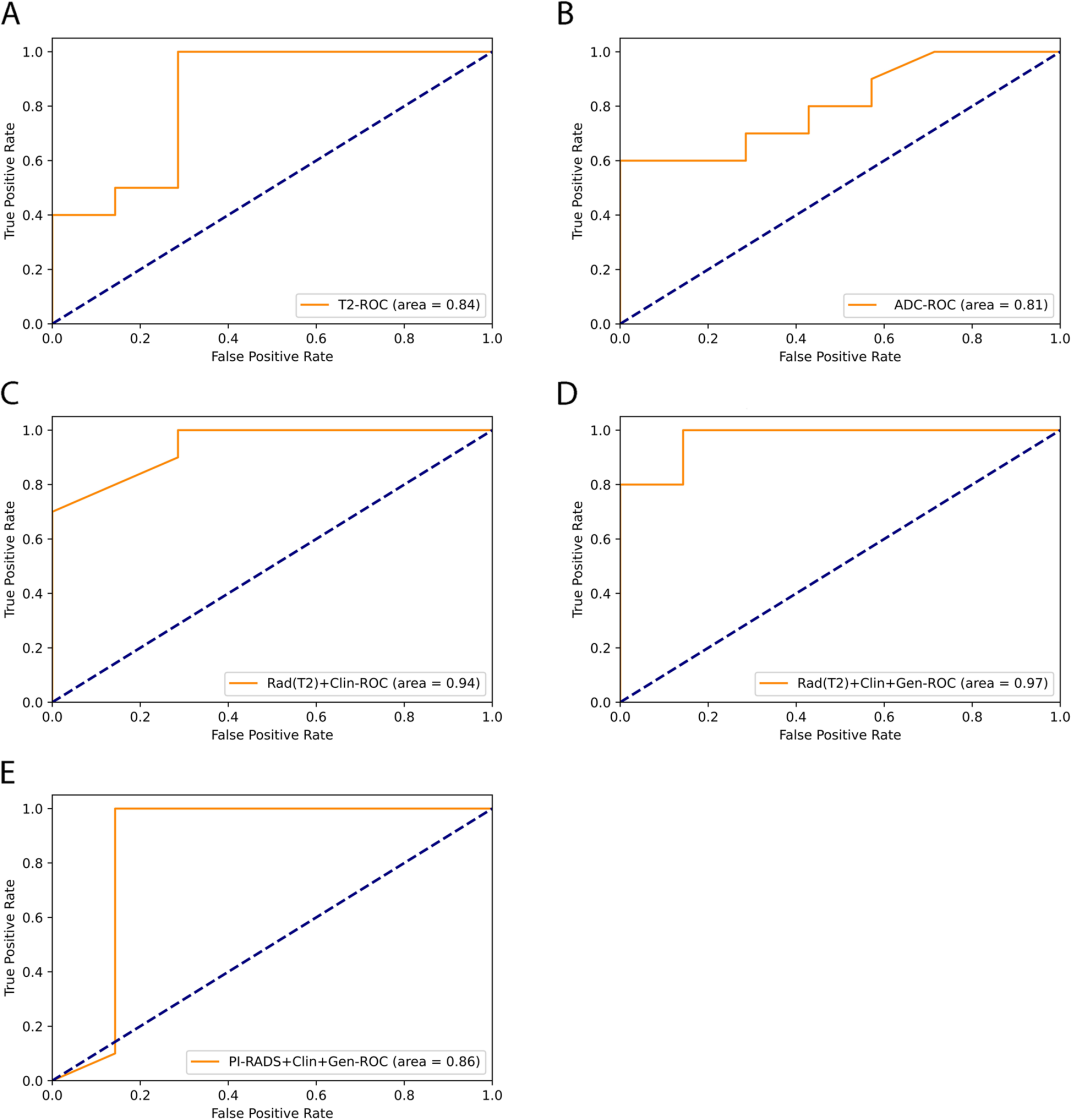
ROC curve of the prediction models. Legend: **A** ROC curve of T2WI; **B** ROC curve of ADC; **C** ROC curve of T2WI combined with clinical data; **D** ROC curve of T2WI combined with clinical data and cg05618150 methylation level; **E** ROC curve of PI-RADS score combined with clinical data and cg05618150 methylation level

**Fig. 6:**
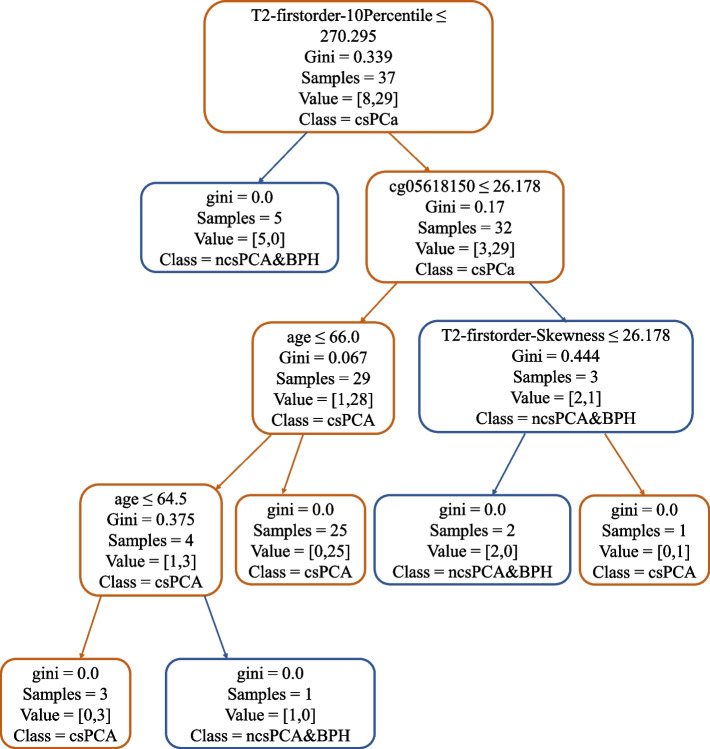
Decision tree of the RF algorithm in the T2WI-clinc-methylation-combined model. Legend: Elements in higher positions had higher priority

The prediction efficiencies of T2WI, ADC, and combined models based on the RF algorithm are shown in Fig. 5. The sensitivity, specificity and accuracy of T2WI were 1.0, 0.71 and 0.88; those of ADC were 1.0, 0.26 and 0.71; those of the T2WI-clinic-combined model were 1.0, 0.71 and 0.88; those of the T2WI-methylation-clinic-combined model were 1.0, 0.71 and 0.88; and those of the model based on the PI-RADS score, the PRKY promoter methylation level (cg05618150) and clinical data were 1.0, 0.43 and 0.76. The T2WI sequence reached an AUC of 0.84, which was higher than that of ADC sequence (AUC = 0.81). The AUC of T2WI-clinic-combined model was 0.94, whereas that of the T2WI-clinc-methylation-combined model was 0.97. With respect to the model based on the PI-RADS score, the PRKY methylation level and clinical data had reached an AUC of 0.86. The decision tree of the T2WI-clinc-methylation-combined model was shown in Fig. 6.

## Discussion

Our study tested the predictive value of PRKY promoter methylation as well as T2WI and ADC sequences, and constructed combined prediction models for csPCA. The T2WI-clinic-combined model exhibited an AUC of 0.94, and the T2-methylation-clinic-combined model reached a higher AUC of 0.97. Previous studies have shown the high predictive value of MRI features for prostate cancer, Liu et al. used multiphase MRI features to predict P504s/P63 Immunohistochemical Expression and reached an AUC of 0.93, Qiao et al. also constructed a prediction model for Gleason grade group with T2 and DWI features, and the AUC of which was 0.92 [24, 25]. The same results were observed in our research, which demonstrated greater efficacy in csPCA diagnosis.

To obtain radiomic images with high quality, a 3.0-T MR was chosen for its increased signal-to-noise ratio which may bring increased spatial resolution compared with a 1.5-T MR [26]. T2WI and ADC sequences were previously reported to be effective in predicting prostate cancer [27]. According to previous studies, T2WI and ADC were negatively correlated with the percentage area of nucleus and cytoplasm, and positively related to the percentage area of lumen space [28]. Lumen space was observed negatively associated with Gleason score, and a more chaotic gland structure was related to a higher Gleason score [29, 30]. Therefore, T2WI and ADC might be considered as relative factors to the Gleason Score. In our study, the results also revealed a strong correlation between the two sequences and a high Gleason grade. The T2WI model has reached an AUC of 0.84 and the ADC model has reached 0.81.

Additionally, the AUC of T2WI was higher than that of ADC in our study, which is different from the findings of several previous studies [25, 31]. However, Liu et al. observed the same phenomenon as in our research [24]. The reason might be related to the following points. First, with a higher resolution ratio, T2WI sequence might provide more details than ADC sequence. Second, T2WI is more efficient in delineating zonal anatomy which is associated with the degree of disorder in the glands [32]. Since the Gleason score system is highly based on the morphological features and arrangement of glands, T2WI might be more relevant to csPCA. Considering that MRI examination at some medical institutions may not include DWI sequences, T2WI was selected for the construction of the prediction model with clinical data and PRKY promoter methylation level. As a result, a high predictive value was observed.

The methylation level of the site cg05163709 on PRKY promoter in urine specimens has already been proven to be associated with Prostate Cancer [15]. However, in our study, the methylation level of cg05163709 was found irrelevant to csPCA in blood samples, but which of cg05618150 was found to be associated with csPCA and have predictive value (AUC = 0.74). Compared with obtaining urine sample, acquisition of blood sample was more comfortable and convenient for patients because of freeing from digital rectal examination. BSP, an efficient method for methylation detection in previous research, was also used in our study to ensure the accuracy [15]. Although the incomplete conversion might affect the accuracy of BSP, compared with other methods for detecting DNA methylation in a single gene, BSP is more convenient and reaches a great sensitivity [33, 34]. After the cg05618150 methylation level was included in the T2WI-clinic-combined model, the AUC of the model has raised to 0.97, which suggested the potential for cg05618150 methylation level to promote predictive value of the radiomic model.

In our study, RF algorithm, the efficiency of which has been tested in previous research, was used for the construction of prediction models with high quality [23]. Although other algorithms including the k-nearest neighbors and the naive Bayes are also effective in processing multiple variables, the RF algorithm based on the decision tress is more convenient to be interpreted by humans [35]. The RF algorithm can process predictors of different scales and distributions and show the relevance of each predictor [36]. Thus, we inputted clinical data include age, PSA level and prostrate volume into the model in expectation of improving the predictive value. To demonstrate the judgement logic of RF algorithm, a specific decision tree was exhibited, which might provide intuitional advice for clinical decision. Interestingly, in the T2WI model, the T2WI-clinic-combined model and the T2WI-clinic-methylation-combined model, the same sensitivity, specificity and accuracy could be observed. This perhaps owes to the high weight of T2WI features in the combined model. Nevertheless, the AUC of the T2WI-methylation-clinic-combined model was still greater than that of any other model in our study, indicating the value of PRKY promoter methylation in the prediction of prostate cancer.

As an important imaging assessment criterion for prostate cancer, the PI-RADS score was excluded when building the predictive model. Our study aimed to construct an objective model to evaluate the predictive value of MRI features. Considering the influence of the subjectivity of radiologists in the process of evaluating the PI-RADS score, only objective imaging features were ultimately included. A model based on the PI-RADS score, clinical data and PRKY promoter methylation was also constructed, but the results showed that the T2WI-clinic-methylation-combined model was more effective than the one with the PI-RADS score. However, considering the difference among radiologists from different hospitals, a multicenter study is still necessary to prove the finding in further research.

The main limitation of our study is that only 54 patients from a single center were included. Thus, selection bias could not be ignored. Second, some clinical features, including body mass index and other biochemical characteristics such as free PSA were not included. Additionally, only BSP was used for PRKY promoter methylation detection. Other methods for sequencing, such as methyl-CpG binding domain-based capture and sequencing or methylation sensitive restriction endonuclease sequencing, might bring different results. Finally, we only constructed the prediction models by means of the RF algorithm. A study comparing various algorithms may help to select a model with the highest predictive value.

Our study constructed a prediction model for csPCa with high predictive value combined with radiomic features, PRKY methylation level and clinical data. Thanks to the convenience in model interpretation of RF algorithm, after further improvement, the model can be exported and processed into clinician-friendly applications that output the possible clinical diagnosis simply after the radiomic features, PRKY methylation levels and clinical data were inputted. This may optimize the diagnostic process of prostate cancer and benefit both doctors and patients.

## Conclusion

In conclusion, our study revealed the association between the methylation level of a site, cg05618150, on PRKY promoter in blood samples and csPCA, tested the predictive value of T2WI and ADC sequences for csPCA diagnosis, and constructed a T2WI-methylation-clinic-combined model with high prediction efficiency via machine learning methods.

## Data Availability

No datasets were generated or analysed during the current study.

## Abbreviations

PCa: Prostate cancer
ISUP: International Society of Urological Patholog
ncsPCA: Non-clinically significant prostate cancer
BPH: Benign prostatic hyperplasia
csPCA: Clinically significant prostate cancer
MRI: Magnetic resonance imaging
PI-RADS: Prostate imaging reporting and data system
PVP: Photoselective vaporization of the prostate
BSP: Bisulfite sequencing polymerase chain reaction
qPCR: Quantitative real-time polymerase chain reaction
Ct: Cycle threshold
T2WI: T2-weighted imaging
FA: Flip angle
TR: Repetition time
TE: Echo time
NEX: Number of excitations
FOV: Field of view
DWI: Diffusion-weighted imaging
ADC: Apparent diffusion coefficient
GLCM: Gray-level cooccurrence matrix
GLRLM: Gray-level run length matrix
GLSZM: Gray-level size zone matrix
NGTDM: Neighboring gray tone difference matrix
RF: Random forest
ROC: Receiver operation characteristics
AUC: Area under the receiver operation characteristics curve
PSA: Prostate specific antigen
ΔCt: The difference in cycle threshold between the sample and control
